# End-to-End Implementation of a Convolutional Neural Network on a 3D-Integrated Image Sensor with Macropixel Array

**DOI:** 10.3390/s23041909

**Published:** 2023-02-08

**Authors:** Maria Lepecq, Thomas Dalgaty, William Fabre, Stéphane Chevobbe

**Affiliations:** Université Paris-Saclay, CEA, List, F-91120 Palaiseau, France

**Keywords:** smart imagers, macropixel array processors, embedded artificial intelligence, convolutional neural networks

## Abstract

Three-dimensional-integrated focal-plane array image processor chips offer new opportunities to implement highly parallelised computer vision algorithms directly inside sensors. Neural networks in particular can perform highly complex machine vision tasks, and therefore their efficient implementation in such imagers are of significant interest. However, studies with existing pixel-processor array chips have focused on the implementation of a subset of neural network components—notably convolutional kernels—on pixel processor arrays. In this work, we implement a continuous end-to-end pipeline for a convolutional neural network from the digitisation of incoming photons to the output prediction vector on a macropixel processor array chip (where a single processor acts on group of pixels). Our implementation performs inference at a rate between 265 and 309 frames per second, directly inside of the sensor, by exploiting the different levels of parallelism available.

## 1. Introduction

Convolutional neural network (CNNs) models serve as the basis for a number of important computer vision applications, including classification [1,2], detection [3,4] and segmentation [5,6]. However, CNNs are memory intensive models, and their execution on conventional hardware, such as central and graphics processing units (CPUs; GPUs), can often result in high latencies and energy requirements. For the most part, this is due to the time and energy required simply to move sensor data, model parameters and intermediate network states between shared memory and processing units rather than performing the underlying calculations themselves [7]. In order to improve the efficiency of CNNs, different hardware paradigms have been developed with the aim of massively reducing the volume of information movement. Numerous dataflow architectures have been proposed [8,9,10,11,12,13,14], whereby an array of processing elements (PEs), each containing their own limited memory called a register file, minimise the flow of data by storing intermediate results locally as well as operating on data received from their neighbouring PEs. Similarly, tensor processing units have been proposed which accelerate matrix multiplication through the cascading of multiplication and sum over a systolic array of PEs [15].

While these approaches allow neural network models to execute more efficiently once the input data are in place, for computer vision tasks, there remains the cost inherent to transporting the input pixel data from an image sensor to a standalone processor. To solve this problem, imager chips have been proposed that integrate processing elements with the pixels themselves [16]. Such approaches, called pixel-arrays, allow computer vision algorithms to be executed inside of the sensor while also storing neural network weights and intermediate data inside the register file of the pixel processors. This in-sensor approach promises a considerable reduction in the energy required to perform inference in embedded systems at the edge [17].

Three-dimensional-integrated imager chips, whereby the photodetector array is stacked directly on top of a processing layer, offer considerable advantages in terms of pixel density and low latency processing [18,19,20]. Integrated solutions have been also proposed where one die, that performs a complete image capture, is stacked on top of another which executes the digital signal processing steps required for CNN inference [21]. However, since the data connection between the two dies relies on a column analog-to-digital converter, the bandwidth between the sensing and processing components may be limited. By connecting pixels, or sets of pixels (i.e., macropixels), directly to the processing array, this limitation can be overcome.

The previous work has demonstrated advantages in terms of energy, latency and frame rate, of implementing convolutional neural network layers on pixel array chips [22,23]. However, while pixel processing arrays offer highly parallelised processing, the hardware computing capabilities of the circuits below the pixels may be constrained by the size of the pixels themselves. Macropixel processor arrays mutualise hardware resources per set of pixels and therefore offer a compromise between the processing parallelism and hardware capabilities, allowing more complex processing that cannot be conducted at the pixel level.

In this paper, we present the first end-to-end implementation of a convolutional neural network model on a macropixel processor array (MPA) chip. We compare the frame rate achieved on our architecture to previous works based on pixel-arrays. We achieve a favourable performance for neural network architectures of similar sizes but with higher precision weights and activations.

## 2. Materials and Methods

### 2.1. Three-Dimensional-Stacked Macropixel Array Architecture

**Figure 1:**
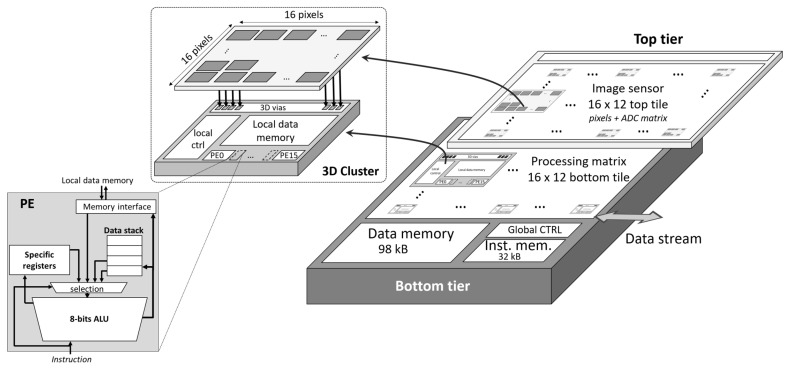
Three-dimensional-stacked macropixel processor array overview: (**top left**) 3D-stacked macropixel array with 16 × 16 pixels tightly coupled to a SIMD of 16 PEs (**bottom left**). Details of one PE (**right**). A full matrix of MPX-p.

This work is built on the RETINE [18,19] MPA. It is a 3D-stacked vision chip whereby an array of backside-illuminated photodetectors are bonded directly on top of an array of macropixel processors (MPX-p) (see Figure 1). Specifically, an array of 16 × 16 photodetectors communicate vertically via sixteen analog-to-digital converters and write the sensed data directly into a local register file (RF) memory that exists within each macropixel processor (MPX-p)—each with a total capacity of 384 bytes. Each MPX-p also contains sixteen local processing elements (PEs) that execute programmed microcodes in a single-instruction multiple-data fashion. These microcodes are generated through compiling a custom assembly language. The sixteen PEs write into and read from one of sixteen corresponding columns in the RF in parallel and perform logic and arithmetic operations on these data (i.e., add, multiply, shift, etc.) thanks to an 8-bits arithmetic and logical unit (ALU). Furthermore, RF data can be shifted in parallel, to the left or right, between data columns within the register file and also between neighbouring in any direction. This local MPX-p memory can also be written to and read from by an on-chip 98 KB SRAM in a highly parallel and low latency fashion via a crossbar circuit. This might be conducted, for example, to load the weights of a convolutional kernel that will be applied to the pixel data stored in the RF.

These 3D-stacked MPX-p are tiled in a 16 × 12 systolic array—permitting massively parallel and distributed in-sensor computation on 256 × 192 pixels captured by the top layer of photodetectors on the chip at a rate of up to 5500 frames per second. RETINE can also be configured to operate in a higher image resolution mode of 1080 × 768 pixels.

### 2.2. Convolutional Neural Network Model

Very similar to the LeNet-5 convolutional neural network [24], the neural network architecture applied in this work relies on convolutional and fully connected layers. More precisely, the model has two convolutional layers followed by two fully connected layers, as summarised in Table 1. Since the main objective of this paper is to study and detail the implementation details of a CNN on an MPX-p, we therefore consider the arbitrary use case of MNIST digit classification.

An input image of dimension 24 × 24, at a fixed location within the full field of view, is fed into the model which outputs a vector of size ten, denoting the class (i.e., digits 0 through 9) logits pertaining to the input digit. After each convolutional and fully connected layer, the weighted sum is summed with a bias-shared parameter that is a common value for each layer, and then passed through a saturating rectifying linear unit (ReLU)—-the saturation value is fifteen. A bit-shift scaling factor *N* is introduced into the ReLU operation applied to the weighted sum per layer. This acts to divide the weighted sum by a factor of $2^{N}$, such that the distribution of weighted sums over the training dataset falls within the permitted range of activations between zero to fifteen [25].

In order to respect the memory constraints of the RETINE MPA, the input image is binarised and, in a post-training quantisation step, the weights and activations are quantised to 4 bits (twos-complement signed integer for weights and unsigned integers for the activations) after training. Note that our implementation supports an intermediate precision in the weighted sum of up to the 17-bit signed integer precision. At this level of quantisation on RETINE, the accuracy on the MNIST digit test set is reduced by only 1.4% compared to an 8-bit quantisation—from 98% to 96.6%—and the resulting 34 kB neural network can be fully stored in the MPA on-chip SRAM.

## 3. End-to-End Implementation on the MPA Architecture

The neural network is implemented on RETINE by a series of microcodes that execute the operations required to implement the four layers of our model in a continuous pipeline. In addition to the functional codes that implement the required core operations (convolutions and fully connected operations), pre-processing codes are also required before each layer in order to organise and distribute the data (input features (IF) and weights) over the MPA architecture to take advantage of all levels of parallelism available.

**Figure 2:**
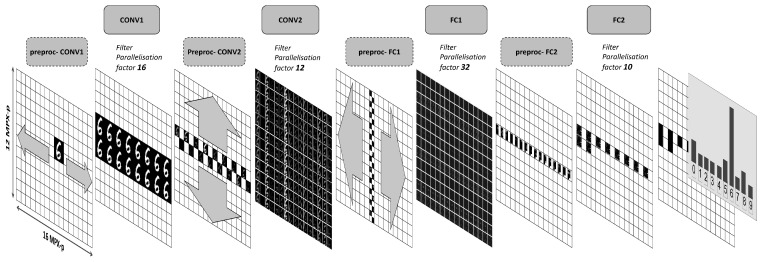
End-to-end pipeline implementation of the convolutional neural network model on the MPA. Each intermediate state of the data from input digit to output class distribution prediction is represented on the 16 × 12 array of MPX-p. Grey arrows indicate the flow of data over the array. The extent of filter parallelism for each layer is noted in the upper part of the figure.

We first detail the end-to-end pipeline, as depicted in Figure 2, and then in the following section provide more detail relating to how the core functions (i.e., convolution, multiply and accumulate) are realised. All of the intermediate feature maps and vectors shown in the figure correspond to those which resulted on the MPA due to the input digit six shown in the figure.

### 3.1. High-Level Pipeline

**Figure 3:**
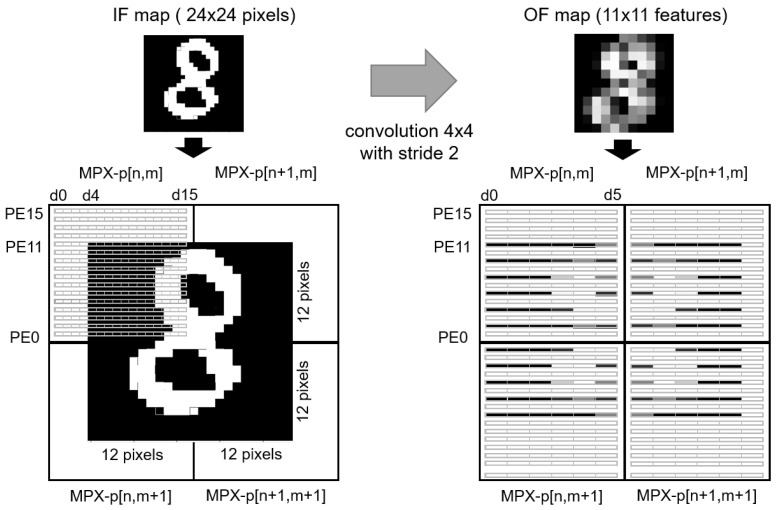
Example of how the input and output feature maps are distributed over four MPX-p in the CONV1 layer. The left-hand side image shows the input MNIST digit spanning four MPX-p.

The entire field of view, transduced by the top-layer of the 3D-stacked image sensor, is binarised in a low resolution mode (256 × 192 pixels) by the analog-to-digital converters and written into the local register file of each MPX-p. Assuming that the region of interest (ROI), in other words, the input MNIST digit, is in the centre of the field of view, the 24 × 24 input binary pixels of the digit are stored in a square spanning four MPX-p— each observing a 12 × 12 pixel quadrant of the MNIST digit (Figure 3).

#### 3.1.1. Pre-Processing CONV1

In order to apply the sixteen filters of the first convolutional layer (CONV1) in parallel, the input ROI is duplicated fifteen times on the MPA. This is conducted first by copying the ROI data to the four southerly MPX-p. The two central MPX-p columns containing the ROI are then duplicated over the width of the MPA. After duplication of the input, the weights for the CONV1 layer are then loaded into the MPA from the on-chip SRAM. Each square group of four MPX-p, which contain the input ROI, are programmed with the weights of one of the sixteen different convolutional filters.

#### 3.1.2. CONV1

For each filter, the convolution operation is performed in a highly parallel fashion, as represented on Figure 3. Not only is one kernel parallelised over four MPX-p, but twelve of the sixteen available PE with an MPX-p operate simultaneously on by twelve columns of MNIST digit pixels.

The convolutional filter stride is implemented during the convolution. After application of the filters, a ReLU activation is applied and the outputs are quantised as unsigned 4-bit integers—resulting in a 11 × 11 output features (OF) map. The four quadrants of each of the sixteen OF maps are regrouped into one of the four MPX-p, as in the third panel of Figure 2. All CONV1 layer filters are executed in parallel, and at the end of this layer the sixteen OF maps are distributed over the two central lines of the MPA (cf. Figure 2).

#### 3.1.3. Pre-Processing CONV2

The CONV1 OF maps are then duplicated vertically, such that each column of the entire MPA contains one OF. This is achieved in two steps. In the first, the two central rows of MPX-p are filled with the OF. Then, in a highly parallelised fashion, data from the first line of MPX-p are transferred to the upper part of the matrix, while data from the second line are transferred to the lower part.

At this point, the CONV2 layer inputs are duplicated twelve times (on the twelve MPA rows). Crucially, this permits the simultaneous execution of the twelve filters. Since the second convolutional layer requires twenty-four filters, the kernels can be applied in two passes. Before each pass, one set of filter weights are loaded into the MPA, such that each MPX-p contains twenty-five weights—corresponding to the 5 × 5 kernel of a channel for a filter. Each row of MPX-p are loaded with the same filter weights. In each row, therefore, one filter is applied to all of the feature maps from the previous layer.

#### 3.1.4. CONV2

The convolutions for the first twelve filters are executed on the MPA. In one row of the MPA, each MPX-p contains the 11 × 11 input feature map corresponding to a channel and each PE from $PE_{0}$ to $PE_{10}$ contains in its local register file, a line of eleven IF. Similarly to CONV1, the filter is applied simultaneously, in parallel for each set of filters and for the set of outputs from the previous layer, on eleven of the sixteen PE for the eleven columns of the input feature map. Weights for the last twelve filters of the CONV2 layer are then loaded into the MPA before executing the next set of convolutions for the last twelve filters.

**Figure 4:**
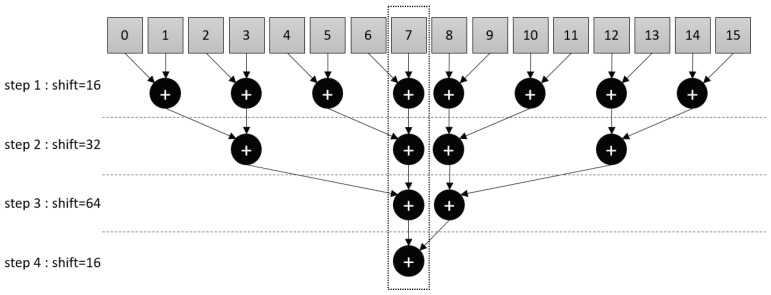
The addition tree execution steps for a row of sixteen MPX-p. Numbered grey squares correspond to MPX-p in a row of the MPA. Arrows converging on addition symbols (within a black circle) show how data from these MPX-p are summed together spatially. After four branches of the tree, the final results converge at the central MPX-p column (here MPX number 7). The communication shift value is specified for each branch at the left-hand side of the figure.

At this point, each MPX-p contains a partial output feature map. In order to complete the convolution, a weighted sum of all of the feature maps in each row of the MPA is required. This is achieved by executing an addition tree microcode (Figure 4). Simply, the addition tree sums up, along the rows of the MPA, all of the partial results stored in each MPX-p (i.e., for each channel). It is executed in parallel for the twenty-four filters whereby the sum converges, over four addition branches, towards a central column of MPX-p. Between each addition branch, the partial sums are bit-shifted between neighbouring MPX-p, where a different number of shifts are performed at each step. The final 17-bit signed weighted sum is then finally passed through a ReLU activation and quantised to 4-bits. At the end of this layer, the twenty-four 4 × 4 OF maps are located in the MPA central column as represented in Figure 2. Within each MPX-p, the data are organised such as that the feature maps are contained within the memory of eight of the PEs.

#### 3.1.5. Pre-Processing FC1

To parallelise the calculation of the 150 filters of the FC1 layer, IF maps are first duplicated once inside the MPX-p: features located in the first eight PEs are copied into the eight remaining PEs ($PE_{8}$ to $PE_{15}$), thereby doubling the number of partial results that can be evaluated inside a single MPX. The central column of the MPA is then horizontally distributed such that each row in the MPA contains copies of the same output feature map from the previous layer.

#### 3.1.6. FC1

At this point, each column of the MPA contains the flattened 384 ReLU activation vector from the CONV2 layer. Each MPX-p will compute the partial weighted sum for two hidden neurons in a single pass—therefore, thirty-two neurons on the entire MPA. Each MPX-p is programmed with sixty-four 4-bit signed weights, corresponding to a 32 × 2 weight matrix. To compute the partial products for the 150 hidden layer neurons, five passes are therefore required. After these five passes, to compute the full weighted sum, the partial products stored in each MPX-p are summed vertically over the MPA using another addition tree code. This sum converges in a central row of the MPA, as shown in Figure 2. A ReLU activation is then applied and the output is reduced from a vector of 16-bit signed integers to 4-bit unsigned integers.

#### 3.1.7. Pre-Processing FC2

The entire vector of 150 4-bit ReLU activations from FC1 layer is distributed over the sixteen MPX-p of the central row in the MPA. In preparation for the final fully connected layer, all the activation values are regrouped into one MPX-p, which is then copied to nine others to allow the computation of the ten final output neurons in parallel. Each of these MPX-p are loaded with 150 4-bit signed weights.

#### 3.1.8. FC2

The entire calculation executes within a single MPX-p and no further communication with neighbouring MPX-p are required. The single value calculated per MPX-p corresponds to the output logit of that neuron. The output class distribution for the example input digit, calculated on the MPA implementation, is shown in the final stage of the pipeline in Figure 2.

### 3.2. Description of Core Functions

In the pipeline described above, there are two core functions that underpin the neural network model: convolution operations and vector–matrix multiplications (i.e., fully connected layers). These operations are well known and widely used on CPU and GPU architectures, but their efficient implementation on a specific architecture such as a highly parallel MPA architecture is a challenging task. One of the main issues is to exploit the computing power in parallel without adding too much data movement time. The following section details, from the perspective of register file of an MPX-p, how these operations are implemented to exploit the MPA parallelism.

#### 3.2.1. Convolution

**Figure 5:**
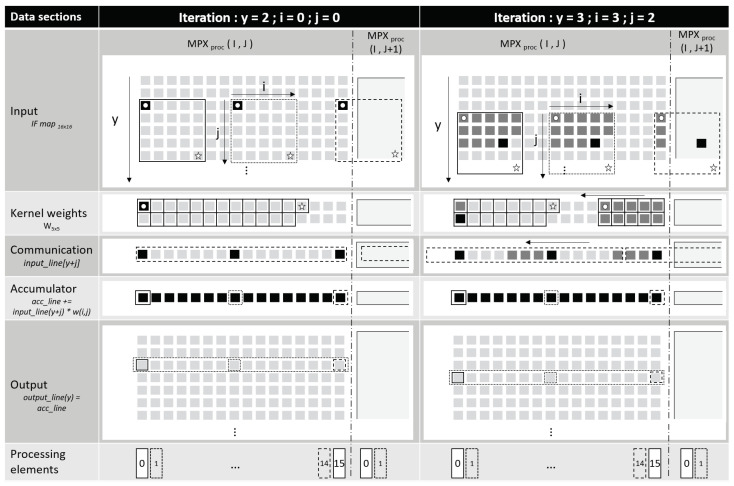
Convolutional kernel implementation in a MPX-p. Two example states of a 5 × 5 kernel convolution at y = 2; i = 0; j = 0 and y = 3; i = 3; j = 2. Input, kernel weights, communication, accumulator and output data section are represented as rows of a table. An additional bottom row shows the PEs that operate on the columns of data in the register file. Each grey or black square corresponds to a data stored in the register file. Black squares identify the current data in use by a processing element and dark grey squares show data already processed on the current Y iteration by the kernel. White circles and stars in the input and weights sections mark the start and end points of the operation.

The local register file memory of each MPX-p can be viewed as depicted in the two panels of Figure 5 (each panel shows a different state of the RF at a different moment in the convolution). The RF memory is divided into five sections. The first section, input, contains the data on which the convolutional filters are applied (i.e., pixel data or input feature maps); the second, kernel weights, is loaded with the filter weights; and the third, communication, is used for communicating input data between neighbouring columns of PE and neighbouring MPX-p. The fourth section, accumulator, contains the accumulated values of an ongoing convolution, which, after the operation is finished, are written into the fifth section, output, that contains the output feature maps of the convolution.

As described in Section 2.1, each PE within an MPX-p operates on one corresponding data column in the RF. Each of the sixteen PE applies the filter (loaded into the second section) simultaneously, while the data values are transferred through the communication section to be multiplied by the corresponding weight in the PE. The convolution exploits a specific feature of the MPX-p, whereby the weight value currently contained within the zeroth column of the register file (the leftmost in Figure 5) can be broadcast simultaneously to all sixteen PE. Concretely, the input data value loaded into each PE is multiplied by the broadcast weight value and, in order to cycle through all weights of the loaded filter, the weights are repeatedly shifted from right to left within the register file. By copying the value in column zero into column fifteen, the weights circle indefinitely within an MPX-p.

The convolution mechanism is composed of three nested loops (denoted by arrows in the input section of Figure 5): loop y, which loops over each row of data in the input data section, and the kernel loops i and j—where I and J correspond to the dimensions of the convolutional kernel (both equal to five in this example). Three dashed boxes in Figure 5 show the neighbourhood over which a subset of three PEs apply the kernel. Black squares denote the current point of execution within the nested i and j loops, while grey squares show the data points that have already been multiplied by the corresponding weight in the weight section. At the beginning of each i loop, the (y + j)th row of input data is loaded into the communication section. After each multiplication in the i loop, the communication section shifts its contents progressively from left to right. Note that for processing elements on the right-hand side, input data are shifted in from the neighbouring MPX-p; therefore, there are no edge effects between MPX-p.

The results of the sixteen parallel multiplications are summed with the existing sixteen values in the accumulator section over the i and j loops. At the end of each outer y loop, in other words after applying the kernel to a full row of input data, the sixteen accumulated values are written into the corresponding column in the output section and the accumulator is reset to an initial value—here a learned bias parameter. The weights are also realigned to their original position.

Figure 5 shows two intermediate positions of this convolution operation. In the left panel (y = 2, j = 0, i = 0), the PEs are operating on the data in the same column and the weights are in their initial position. The results of the accumulator after application of the kernel will be written into the second line of the output section. In the right-hand side panel (y = 3, j = 3, i = 2), the PEs are operating on the next row of input data but the kernel is in a more advanced state. Note that the weights section has been shifted several times, wrapping around from the zeroth to the fifteenth PE. Furthermore, the input data that have been loaded into the communication section at the beginning of the third j loop have also been shifted three times to the left.

After iterating over all Y rows in the input data section, the convolution operation has been completed and the output section contains the output feature map. In order to implement a stride on the convolution, two additional steps are required. First, for the vertical component of the stride, it is simply required to skip a certain number of y iterations. To implement the horizontal component of the stride, the values in the feature map are multiplied by zero and then the columns of the output feature map are shifted to the left. The implementation of the convolution operation described above is illustrated for a 5 × 5 kernel but it remains applicable for any kernel size, the only limitation being the space available in the register file.

As detailed in Section 3.1, two convolutional layers are required in the model implemented on the MPA. The first requires the application of sixteen 4 × 4 convolutional kernels to a digit spread over four MPX-p (Figure 3). For this layer, the same set of sixteen weights (one per MPX-p register file column) are loaded to all four MPX-p and perform the convolution on four quadrants of the image in parallel. In the second convolutional layer, composed of twenty-four 5 × 5 kernels, the output feature maps from the first layer are processed in two steps. First, the convolution operation is applied to each channel within a single MPX-p—consistent with the presented operation in Figure 5—resulting in sixteen partial output maps distributed in sixteen MPX-p, which is then followed by the addition tree operation to accumulate the partial results into a single MPX-p.

#### 3.2.2. Vector-Matrix Multiplication

The second core operation, required for the final two layers of the model, is fully connected layers. This requires one simply to compute the inner-product between an input vector and a weight matrix. If the input vector is of a dimension *N* and the desired output vector is of dimension *M*, the weights will be a N×M matrix.

The register file memory within an MPX-p is organised similarly, as in the case of convolution (Figure 5). An input data section, which may be composed of a plurality of rows, stores the input vector. To store, for example, an input vector of sixty-four elements, since there are sixteen PEs, this section would be required to be four rows of data long. A second section contains all of the weights that will be multiplied with this data. The number of rows in the weights section is required to be an integer multiple of the number of rows in the input section, such that there exists *M* sets of weights—one for each of the *M* outputs. For example, if M=4 (i.e., there are four output neurons), a total of sixteen rows are required for this section. Each set of four rows in this section corresponds to the synaptic weights applied to all the input features to give one output feature. The third section is an output data section which has *M* rows—one for each element of the output vector. There is also an additional communications section, used to help calculate the accumulation of the partial product calculated in each PE.

In the execution of a fully connected microcode on an MPX-p, all *M* sets of *N* weights are multiplied with the *N* input data elements. These multiplications are performed in parallel in batches of sixteen—one per processing element. After each multiplication operation, the result is accumulated in the output section. After all *M* sets of weights have been applied to the input, the partial products in each of the PEs must be summed together. This is achieved by performing an addition tree internally within the MPX-p. After the addition tree is completed, one of the PE will contain the full partial result for an MPX-p. In order to compute the full weighted sum between MPX-p, there is an addition tree code that sums partial products from across MPX-p in an MPA.

#### 3.2.3. Addition Tree

Since partial products are computed in a parallelised fashion across the MPA, their elements are required to be summed together in order to calculate the final weighted sum. The addition tree moves data stored in a given RF section to neighboring MPX-p’s, where it is added to data stored in the same section.

The register file memory of each MPX-p is simply divided into two sections. The first section, which is configured to store data up to a 17-bit signed precision, contains the input data to accumulate. The second section is used to communicate between neighbouring MPX-p.

In the loop, the data in the first section are written into the communication section, which is then shifted as many times as necessary to move the data into the communication section of a neighbouring MPX-p. The direction of the communication is configured before the transfer (i.e., North, South, East or West). For example, to reach the nearest neighbouring MPX-p to the left, the communication section is configured for West communication and the data are shifted the appropriate number of steps from right to left. Since a single shift operation moves data between adjacent PEs, and because each MPX-p contains sixteen PE, it is required to shift the data sixteen times to move the contents of one RF section completely between MPX-p. After the section has been shifted, the data are then simply accumulated with the contents of the destination MPX-p by summing it with the data currently in the first section. If the precision of the data is larger than that permitted in the communication section or larger than the PE precision, the data can be cut into slices, and communication-accumulation can be achieved in an incremental fashion.

The addition tree code is repeated over a certain number of addition branches until all of the data distributed over a row or column of MPX-p have been accumulated into a single MPX-p. The first addition branch takes place between neighbouring MPX-p and those that follow with increasingly distant MPX-p in a symmetrical fashion, such that the sum converges towards the center, as represented in Figure 4. The number of shift operations varies in integer multiples of sixteen depending on the distance between two MPX-p that are summed in an addition branch.

As mentioned in Section 3.1, two addition trees are required in the implemented pipeline. The first one—consistent with the Figure 4—accumulates the partial results horizontally of each line of sixteen MPX-p in the CONV2 layer. The second one accumulates the partial results vertically for each of the twelve rows MPX-p in the FC1 layer.

## 4. Results

The above detailed end-to-end pipeline was implemented on the RETINE MPA chip at a clock frequency of 100 MHz. The results and intermediate states achieved on the chip implementation correspond exactly to those observed in the quantised software version of the model.

The latencies, for each pre-processing and functional step in the pipeline, are shown in Table 2. The total time taken to classify an input digit was measured to be 3.8 ms—corresponding to a frame rate of 265 frames per second (FPS). Among all of the steps, the second convolutional layer incurred the largest latency, at 1.6 ms in total, while the second fully connected layer was the fastest to execute—at only 6.1 μs. For the first three layers, the pre-processing steps, responsible for preparing the data and loading the weights into the MPX-p, correspond to between 10% and 20% of the time taken for the following functional step. In contrast, the pre-processing latency required for the second fully connected layer is almost two orders of magnitude greater than the execution time of the layer itself. This is due to the more complex mechanism required to regroup together the results from across the MPA and duplicate these data in ten of the MPX-p, compared to the relative simplicity of the operations required to realise the second fully connected layer.

**Figure 6:**
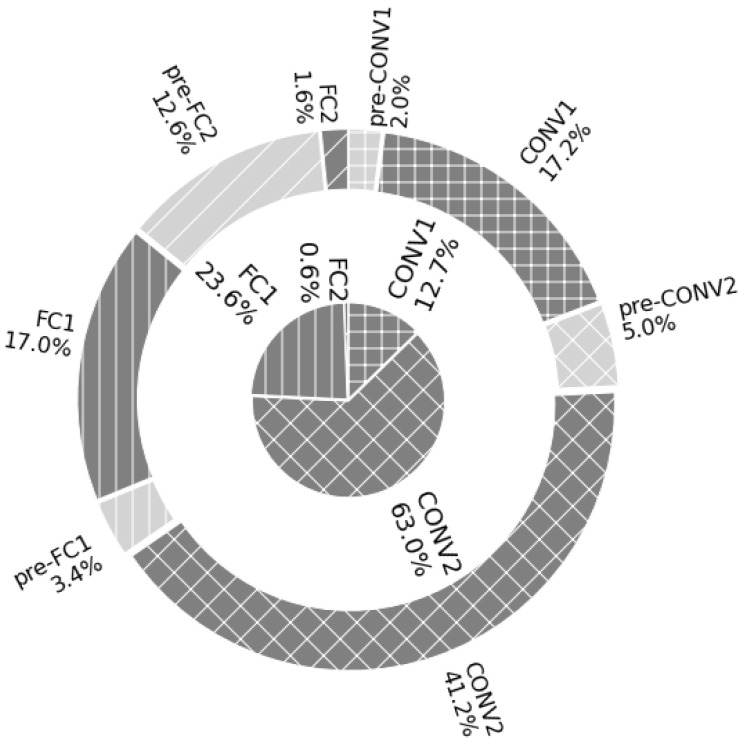
Nested pie charts comparing the (inner) theoretical calculation complexity and (outer) the resulting percentage of the total latency of the layer implemented on the MPA. The pie-chart elapses from zero degrees in a clockwise fashion and different colours correspond to each layer.

In order to better visualise how each step contributes to this total latency, we plot the percentage of the total time of each step in the outer pie chart of Figure 6. An additional inner pie-chart shows the theoretical computational complexity of each layer—simply the total number of additions and multiplications per layer. This comparison offers an insight into the efficiency of the implementation of each layer. What is striking is the imbalance between the theoretical complexity (0.6% of total MACs) of the second fully connected layer and the percentage of the total time taken on the MPA to implement it (14.2% of total latency). As discussed, the latency required to perform the pre-processing for this layer exaggerates this difference even more. In stark contrast, however, the MPA implementations of the second convolutional layer and the first linear layer contribute less to the total time than would be expected from the computational complexity of these layers.

**Figure 7:**
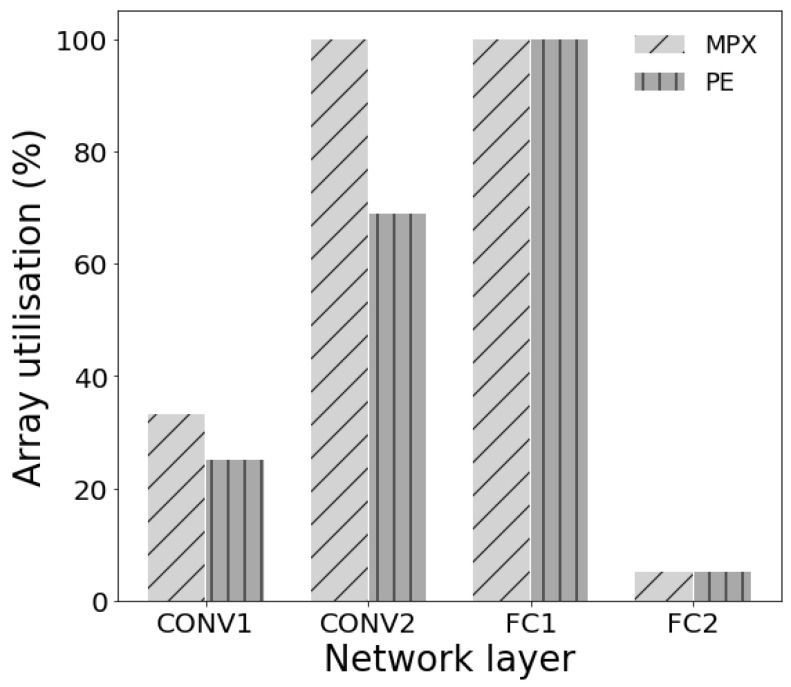
A bar plot comparing the utilisation of MPX-p across the MPA as well as the utilisation of processing elements with the MPX-p for each layer of the model.

This disparity is in large part due to the fashion in which data can be distributed over the MPA (depicted in Figure 2) to permit the maximum parallel usage of the computing resources. The bar chart in Figure 7 shows the percentage utilisation of all available MPX-p and their processing elements (PEs) for each layer. While the second convolutional layer and the first fully connected layer make excellent use of the full array, in particular the first fully connected layer, which leaves no PE untapped, the first convolutional layer and, especially, the second fully connected layer do not. The extent of parallelism is the main reason for the difference between the theoretical and implemented latency observed in Figure 6. In certain applications, instead of implementing the second linear layer on the MPA itself, it might be favourable to offload this computation to an external microprocessor that could handle this calculation faster. In doing this, the pipeline on the MPA would be reduced to 3.2 ms per digit—increasing the frame rate to 309 FPS.

In Table 3, we present a more fine-grained look into the sub-steps that are performed for the entire second convolutional layer to better understand how different operations contribute to the overall latency. After the pre-processing step, already present in Table 2, the next step is the loading of the 4-bit kernel filter weights into the MPX-p. The time taken to load the weights from the on-chip SRAM into the full matrix of MPX-p requires only 390 ns. Using these weights, the MPA applies the first twelve convolution filters—taking a total time of 567.5 μs. Thanks to the highly parallel data transfer mechanism between MPX-p register files and the on-chip SRAM, loading the weights amounts to less than one-thousandth of the time spent computing with them. The second pass requires an additional thirty microseconds since it performs further data manipulation operations that are required to preserve the results from the first pass. The addition tree code, which sums up the partial results of each MPX-p in a tree-structure that converges towards a central column of MPX-p, requires 374.5 μs. Despite its conceptual simplicity, relative to the convolutions, this step requires a series of different microcodes to be loaded into MPX-p across the array (loading a microcode requires around 8 μs each time). The microcode that realises the ReLU that operates on the final feature map requires only 21.9 μs.

Considering the pre-processing step and the weight transfer as data movement steps, only 11% of this total latency is incurred due to data movement operations, while the remaining 89% is due to actual functional computations using this data. In contrast to typical von Neumann-based neural network implementations, our CNN implementation on RETINE succeeds in spending the majority of its time computing with data rather than transporting it.

In this work, we have achieved end-to-end CNN inference with 4-bit precision weights and activations at 265 FPS. As the last fully connected layer is very inefficient due to its lack of parallelism, we can achieve CNN inference at 309 FPS. To our knowledge, the SCAMP-5 [16] pixel-array processor is the only other fabricated pixel-array processor on which end-to-end neural networks have been implemented. We therefore compare our CNN implementation on the RETINE MPA to other implementations of convolutional models on this pixel-array processor in Table 4. Specifically, we look at the number and complexity of layers, the precision of the weights and activations and the resulting frame rate that was achieved, since this offers a reasonable proxy to the efficiency of the implementation. Our macropixel-array-based implementation on RETINE achieves equal or favourable performance on the pixel-array implementations for a similar network size. The fairest comparison can be made between lines two and five of Table 4—both networks realise two convolutional layers followed by a fully connected layer. While the pixel-array has a frame rate of 224 FPS, our macropixel-array-based implementation achieves 309 FPS—a 38% improvement. Furthermore, while the pixel-array uses a 1-bit precision, our macropixel processor array supports weights and activations of up to 4-bits. This may ultimately permit more performant neural networks to be realised—while the 1-bit precision model on the pixel-array obtained accuracies ranging between 92–94%, our implementation achieved a score of 96.6%.

The fact that, for a similar size of CNN, we achieve not only a higher frame rate, but do so with a higher bit-precision, demonstrates the advantage of the macropixel-level calculation over single pixel-level calculation. Since the size of the processor is constrained by the form factor of the pixels above, macropixels allow for a more complex digital circuit and a larger data and microcode memory to exist beneath a group of pixels than is possible under individual pixels.

## 5. Discussion

We have presented the first end-to-end pipeline of a convolutional neural network model implemented on a 3D-integrated general purpose macropixel array chip. The model was implemented with 4-bit precision weights and activations while allowing for intermediate signed mixed-precision calculations of up to 17-bits. Both convolutional and fully connected layers were found to be implemented very efficiently when their weight matrices operated in parallel on different subsets of the input data distributed across the full processing array. However, as parallelisation was reduced, the resulting layer implementations became increasingly less efficient. This was in particular for the second fully connected layer, where parallelisation was difficult due to the small size of the output vector of the layer. While we have implemented a Lenet-based neural network, the MPA is programmable and supports a wide range of neural networks. In this implementation, we have spatially distributed the number of filters as well as the ifmaps over several macropixels in parallel. The number of parallel units for each layer is a trade-off between the memory available locally in the macropixel, and the amount of data to be transferred to perform the processing. The mechanisms used to implement this network can be adapted for more complex networks. However, this scaling will probably be limited by the data movements to feed the computational elements. Fast data transfer mechanisms will probably have to be added.

In a more fine-grained breakdown of the latency incurred within the second convolutional layer, we observed that approximately 11% of the total latency was incurred by data and weight transfer codes, while 89% of the total time was dedicated to processing once the data were in place. Importantly, this demonstrates that computing the in-sensor, in a massively parallel fashion, permits a departure from the von Neumann rhetoric, whereby the majority of the time taken to implement neural network models is consumed due to data movement between either the memory or sensor and processing units.

In comparing our MPA implementation to other pixel-array processor implementations, we achieved a favourable frame rate for similar sizes of the model. Furthermore, we are able to achieve this using a higher bit-precision for the weights and activations. The four layer version of our neural network model implementation on the MPA was able to process 265 frames per second, while for a three-layer version (omitting the final fully connected layer) this was improved to 309 frames per second.

Ultimately, this study has demonstrated that the macropixel processor array-based neural network implementations are advantageous to pixel-array processor arrays because they allow for more operations to be applied per pixel. Macropixels offer a good trade-off between hardware factorisation, computing power and sensor readout rate.

Future works will investigate the implementation of other neural network architectures, for example fully convolutional models with dense output layers (i.e., for detection and segmentation tasks), where the full parallelism of the MPA can be exploited. We will also consider how existing MPA architectures can be revised and how they can be improved using more advanced technology nodes, so that they can support larger and more advanced model architectures.

## Figures and Tables

**Table 1:** Description of the neural network model layers.

Layers	Input Size	Filter Size	Nb Filters	Stride	Output Size
CONV1	24 × 24 × 1 (1b)	4 × 4 × 1	16	2	11 × 11 × 16 (4b)
CONV2	11 × 11 × 16 (4b)	5 × 5 × 16	24	2	4 × 4 × 24 (4b)
FC1	384 (4b)	-	150	1	150 (4b)
FC2	150 (4b)	-	10	1	10 (4b)

**Table 2:** A breakdown of the latency for step of the code. A clock frequency of 100 MHz is used.

Step	Latency (μs)
Pre-processing CONV1	75.5
CONV1	648.9
Pre-processing CONV2	186.9
CONV2	1556.4
Pre-processing FC1	127.9
FC1	641.8
Pre-processing FC2	476.4
FC2	6.1
**Total**	**3774.7**

**Table 3:** A breakdown of the latency for each step required to perform the second convolutional layer. A clock frequency of 100 MHz is used.

CONV2 Step	Latency (μs)
Pre-processing	186.9
Load 1st weights	0.4
CONV first pass	567.5
Load 2nd weights	0.4
CONV second pass	597.1
Addition tree	374.5
ReLU	21.9

**Table 4:** Benchmarking frame rate against convolutional models implemented on the SCAMP-5 pixel-array processor.

Work	Chip	On-Chip Layers	Precision	Frame Rate
Bose 2019 [26]	SCAMP-5	CONV1: 16 5 × 5	2-bit	170
Bose 2020 [23]	SCAMP-5	CONV1: 16 4 × 4 CONV2: 16 4 × 4 FC1: 256 × 10	1-bit	224
Liu 2022 [27]	SCAMP-5	CONV1: 16 5 × 5 CONV2: 128 4 × 4 CONV3: 64 1 × 1	1-bit	283
This work	RETINE	CONV1: 16 4 × 4 CONV2: 24 5 × 5 FC1: 384 × 150 FC2:150 × 10	4-bit	265
This work	RETINE	CONV1: 16 4 × 4 CONV2: 24 5 × 5 FC1: 384 × 150	4-bit	309

## Data Availability

This study uses the following publicly available dataset: MNIST Dataset. This data can be found here: http://yann.lecun.com/exdb/mnist/ (accessed on 1 February 2021).
